# Impact of Climate Change on the Distributional Potential of the Endemic Species *Tamarix dubia* Bunge and Conservation Implications for the Irano‐Turanian Region

**DOI:** 10.1002/ece3.71877

**Published:** 2025-07-28

**Authors:** Habibollah Ijbari, Jamil Vaezi, Maryam Behroozian, Hamid Ejtehadi

**Affiliations:** ^1^ Quantitative Plant Ecology and Biodiversity Research Lab, Department of Biology, Faculty of Science Ferdowsi University of Mashhad Mashhad Iran; ^2^ Herbarium FUMH Ferdowsi University of Mashhad Mashhad Iran; ^3^ Visiting Scholar, School of Environmental Sciences University of Guelph Guelph Ontario Canada

**Keywords:** climate change, ecological niche modeling, endemic species, habitat suitability, MaxEnt model, *Tamarix dubia*

## Abstract

Climate change significantly influences species distribution patterns. Utilizing Ecological Niche Models (ENMs) in climate change research provides valuable insights into species–environment relationships and can inform conservation management decisions. We analyzed climate change effects on the geographic distribution of *Tamarix dubia* Bunge, an endemic species in the Irano‐Turanian region, using ENM approaches. We modeled the current and future suitable areas for *T. dubia* using the Maxent algorithm under two shared socioeconomic pathways (SSP2‐4.5 and SSP5‐8.5) for the period 2041–2060. The results revealed that the mean temperature of the warmest quarter (bio10) and precipitation of the wettest quarter (bio16) were determined to be the most important explanatory climatic variables affecting *T. dubia* distribution. Under both future scenarios, we predicted a decrease in the suitable habitat range of *T. dubia* in the period 2041–2060. Moreover, a relatively high loss of suitability was anticipated in the actual ranges of species. The results indicated that the distribution of *T. dubia* , a drought‐tolerant plant species, is likely to be affected significantly by climate change. This study supports future management plans for *T. dubia* and provides insights into the impacts of climate change on endemic species in arid and semi‐arid regions, which are valuable for understanding distribution patterns and informing future research in the Irano‐Turanian region.

## Abstract 2

Tamarix dubia, a plant species of drought–stress tolerance, undergoes significant climate changes. A relatively high loss of suitability was anticipated in actual habitats of the species using ecological niche modeling approaches. Under future scenarios, a decrease in the suitable habitat range of *T. dubia* was predicted for the period 2041–2060.

## 1 Introduction

Climate change plays a crucial role in the growth and reproduction of plants, as well as in the survival, development, and distribution of species (Kozak et al. 2008; Stephens et al. 2016; Behroozian et al. 2020; Khanal et al. 2022). Future climate change will lead to the fragmentation of suitable areas for species, potentially resulting in declining habitat status (Li et al. 2015). In recent years, a number of studies have indicated that climate change could alter the current habitat suitability and spatial distribution patterns of many species and even lead to the contraction and extinction of some species (Feng et al. 2021; Shi et al. 2022; Varela et al. 2022; Wu et al. 2022). However, some species may benefit from changing climates, expanding into newly suitable areas or shifting ranges in response to warming trends (Behroozian et al. 2020; Karami et al. 2022). As such, predicting the potential distribution of species under global climate change, whether expansion or contraction, is essential in integrative conservation planning for biodiversity (Cavaliere 2009; Anderegg et al. 2015; Karami et al. 2022).

Ecological niche models (ENMs) have been widely used to predict the potential distribution of species under climate change (Elith and Leathwick 2009). ENMs can estimate the potential distribution of species across an area of interest by the mathematical relationship between known species occurrence records and the corresponding environmental variables (Elith and Leathwick 2009; Peterson et al. 2011). These models estimate the suitable spatial distribution for species across the study area and assess the impact of climate change on species distribution on both terrestrial (Kumar 2012; Anderegg et al. 2015) and marine species (Jones et al. 2013). Maximum entropy (MaxEnt) has been developed as a strong tool for modeling species and ecological niches (Phillips et al. 2006). It has been widely applied to predict potential distributions and environmental suitability for different groups; especially, endemic, rare, and threatened species (Phillips et al. 2004, 2006; Qin et al. 2017; Noedoost et al. 2024).

*Tamarix* L., commonly known as tamarisk, belongs to the Tamaricaceae family with ca. 60 species worldwide, mainly distributed in the Mediterranean region and Central Asia (Baum 1978; Qaiser 1976; Akhani 2006). It is a perennial shrub or small tree with scalelike leaves. *Tamarix* species are drought‐tolerant and adaptable to various harsh environmental conditions, including high temperatures and soil salinity (Brotherson et al. 1986; Brotherson and Field 1987; Marlin et al. 2017). Furthermore, these species are known as ecosystem engineers for controlling erosion, acting as windbreakers, and serving as traditional herbs used in medicine to treat infections, wounds, liver, spleen, and gastrointestinal disorders, diabetes, and dental problems (Xia et al. 2020; Bahramsoltani et al. 2020). Given the properties of strong salt and drought resistance, *Tamarix* species can play an important role in maintaining ecosystem stability in saline lands. Therefore, these species can be of unique scientific interest and ecological importance.

*Tamarix dubia* Bunge is a drought‐tolerant species endemic to Irano‐Turanian (Akhani 2006). The native range of this species extends from central and south Iran to southwestern Afghanistan (Rechinger 1970; Assadi 1989). It is a perennial shrub with pale and scalelike leaves and racemes of pink flowers (Rechinger 1970). The scientific evidence of ranges, biosystematics, and ecological information of this species is lacking in Iran. However, a recent study by Ijbari et al. (2024) revealed low genetic diversity in *Tamarix dubia* populations, which could reduce the species' ability to adapt to new environments and make it less capable of colonizing new areas due to reduced resistance to disease, environmental changes, and competition. Based on field observations by the first author and the research of Ijbari et al. (2024), *T. dubia* is currently undergoing population decline and fragmentation due to severe drought in recent years and climate change impacts on natural river flow regimes. Based on this knowledge, *T. dubia* can act as an ideal model species for studying the impact of climate change on species distribution in the Irano‐Turanian region.

Here, we used ENM to assess potential impacts of climate change on the geographic distribution of suitable conditions for *Tamarix dubia*. Accurate knowledge of the species' geographic distribution and the identification of key areas for conservation, such as regions with high habitat suitability, ecological importance, or vulnerability, are critical for informed decision‐making in conservation (Elith and Leathwick 2009; Peterson et al. 2011). The findings from our research are vital for surveying and prioritizing new populations, identifying gaps in the existing conservation network, and guiding the establishment or expansion of protected areas. *Tamarix dubia* is currently not listed on the IUCN Red List, and its conservation status has not been formally assessed in Iran. Furthermore, the species is not included in any recognized national or international conservation frameworks. Given its restricted range, ecological significance, and projected habitat loss due to climate change, an urgent evaluation of its conservation status is warranted, along with its consideration in regional conservation planning. Accordingly, we assessed the effects of climate change on the potential distribution of *T. dubia* in the Irano‐Turanian region by: (1) modeling suitable conditions for the species under current and future climate scenarios, (2) identifying the major environmental parameters influencing habitat suitability, (3) evaluating how projected future climate conditions will affect habitat suitability, and (4) identifying areas for protection and recommending them as new or expanded protected areas. This initial assessment provides valuable insights to guide conservation efforts for *T. dubia* in the Irano‐Turanian region.

## 2 Materials and Methods

### 2.1 Study Area and Data Collection

**FIGURE 1:**
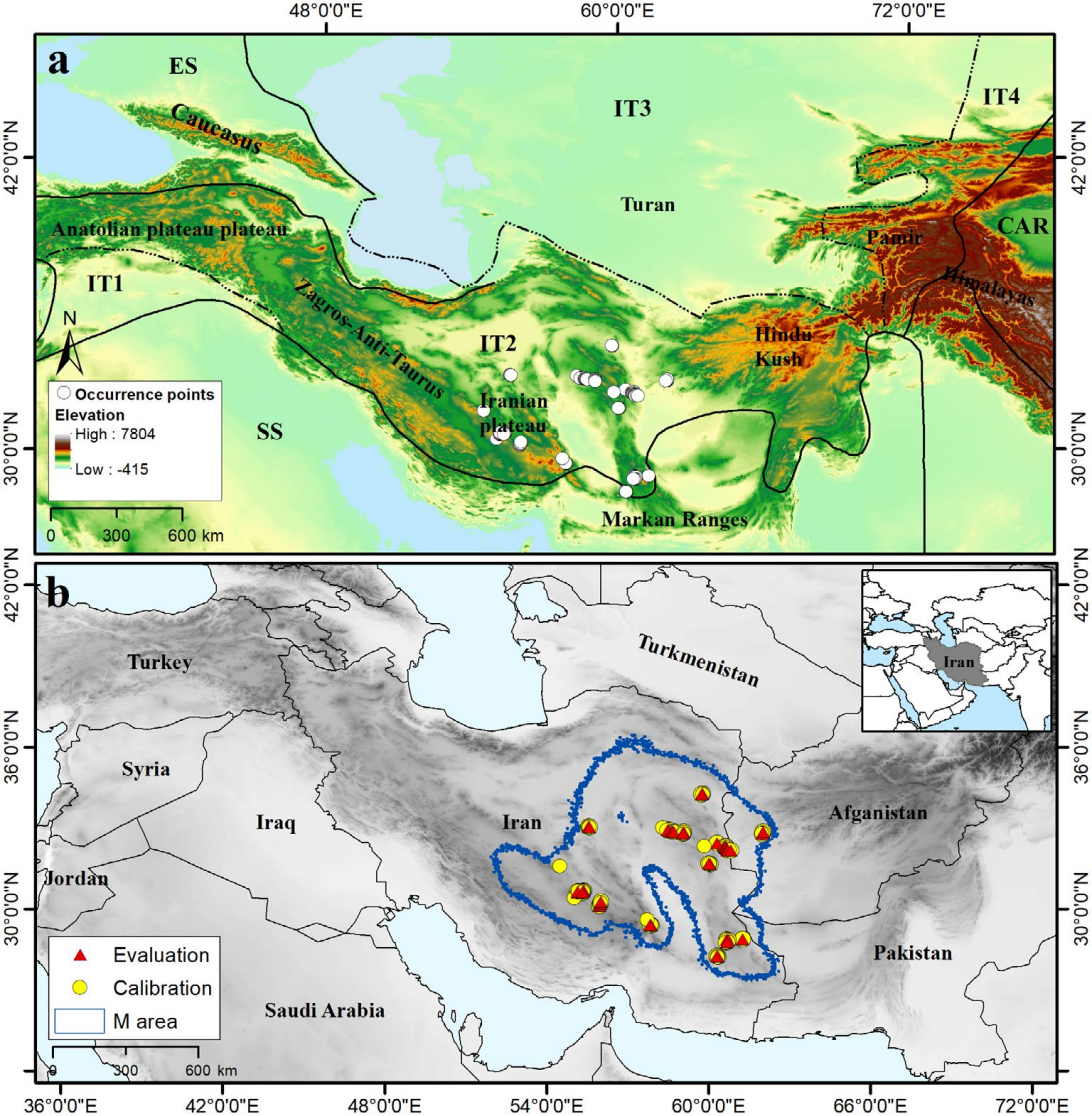
Study area and distribution of *Tamarix dubia*. (a) Phytogeographic subdivisions of the Irano‐Turanian (IT; IT1: Western regional subcenter, IT2: Central subcenter, IT3: Northern subcenter, IT4: Eastern subcenter), Euro‐Siberian region (ES), Saharo‐Sindian region (SS), and Central‐Asiatic (CAR). Phytogeographical borders are taken from Léonard's (1988–1989) (ArcGIS version 10.3.1; http://www.esri.com). (b) Occurrence records of *T. dubia* in a broader view, with the accessible area (M) for model calibration.

We focused on the Irano‐Turanian region (IT region) in southwestern Asia. This region is considered one of the most important phytogeographic zones globally, due to its exceptionally high species richness and endemism, and is divided into four subregions (IT1, IT2, IT3, IT4) (White and Léonard 1991). The subregion IT2 includes many *Tamarix* species. The IT2 subregion, part of the central Irano‐Turanian (IT) phytogeographic zone, is a major center of endemism and botanical diversity in southwestern and central Asia (White and Léonard 1991; Djamali et al. 2011). It encompasses much of central and eastern Iran, including parts of the provinces of Yazd, Kerman, South Khorasan, and Sistan and Baluchestan, where *Tamarix dubia* is primarily distributed (Rechinger 1970; Assadi 1989). IT2 has a distinct climate with low annual precipitation in winter and high in summer, low winter temperatures, and a high Continentality index (Djamali et al. 2011, 2012). The topography of this region is varied, ranging from lowland desert basins to midelevation plateaus and mountains, typically spanning elevations from ~500 to over 2000 m above sea level (Djamali et al. 2012). Soils in this subregion are predominantly aridic and often saline or alkaline, with poor organic matter content—conditions well suited for drought‐ and salt‐tolerant species such as *Tamarix* (Brotherson and Field 1987; Akhani 2006). The region's flora is adapted to harsh, xeric conditions, including large temperature fluctuations and seasonal drought (Zohary 1973; Djamali et al. 2011). Phytogeographically, IT2 represents a transition zone where elements from the Saharo‐Sindian and Euro‐Siberian regions mix with the endemic‐rich Irano‐Turanian flora, resulting in a high rate of species turnover and localized speciation (White and Léonard 1991; Djamali et al. 2011). The vegetation is typically composed of steppe and semidesert communities dominated by xerophytic shrubs, halophytes, and scattered patches of open woodland (Zohary 1973; Akhani 2006; Memariani et al. 2016). This ecological complexity makes IT2 a critical zone for studying the impacts of climate change on narrowly distributed, stress‐adapted plant species (Behroozian et al. 2020; Karami et al. 2022; Noedoost et al. 2024; Ijbari et al. 2024). *Tamarix dubia*, as an endemic plant species in the IT2 region, is distributed in the areas of the center and east of Iran, including five provinces of Sistan and Baluchestan, Kerman, Yazd, South Khorasan, and Razavi Khorasan. It also occurs in a small area of Farah province in western Afghanistan (Figures 1a and S1, Table S1).

Comprehensive field surveys were conducted by the first author at sites throughout the study area. A total of 23 sites were randomly sampled from four geographical regions in Iran: Sistan and Baluchestan, Kerman, Yazd, and South Khorasan, during the 2022–2023 period. One hundred sixty‐three primary occurrence records were obtained for *T. dubia* from these field surveys (140 records) and additional records from previous studies, including literature sources and herbarium specimens. We used Flora Iranica (Rechinger 1970) (five records) and Flora of Iran (Assadi 1989) (eight records). We accessed herbarium specimens in the collections of the Ferdowsi University of Mashhad Herbarium (FUMH) (six records). Occurrence records were also checked from the Global Biodiversity Information Facility (GBIF; http://www.gbif.org/) (five records) and SpeciesLink (http://splink.cria.org.br/) (0 records).

We mapped all occurrence records using ArcGIS version 10.3.1 to assess data quality and removed records with duplicate coordinates, spatial uncertainty greater than 1 km, or incomplete geographic or taxonomic information (Zizka et al. 2019). Then, we eliminated one of each pair of records falling within single grid cells (~1 km) using the spThin library in R Statistical package 3.5.1 (Aiello‐Lammens et al. 2015) to avoid problems with spatial autocorrelation. The final cleaned occurrence data were randomly divided into two equal parts: 50% for model training and 50% for testing. This equal split was chosen to ensure a balanced evaluation of model performance, particularly given the moderate number of occurrence points, and to reduce the risk of overfitting in the MaxEnt framework (Figure 1b).

### 2.2 Climate Data

Initially, we employed bioclimatic raster layers from CHELSA (Karger et al. 2017, 2021; https://doi.org/10.16904/envidat.228) at a spatial resolution of 30″ (~1 km) to develop species distribution models. However, the predicted suitable areas showed poor concordance with known occurrence records, and the resulting maps were highly fragmented, displaying abrupt shifts in habitat suitability that did not align with recognized environmental gradients. Consequently, we adopted WorldClim version 2.1 (http://worldclim.org; Fick and Hijmans 2017), also at a 30″ resolution, for modeling under current climatic conditions. Unlike CHELSA, which emphasizes orographic predictors (e.g., wind fields, valley exposure) and is optimized for complex mountainous terrains (Karger et al. 2017), WorldClim relies more heavily on interpolated weather station data using thin‐plate splines (Fick and Hijmans 2017). In data‐sparse regions such as the Irano‐Turanian zone, which are dominated by arid and semi‐arid plateaus with relatively smooth environmental gradients, the interpolation scheme used by WorldClim tends to provide more stable and continuous climatic surfaces (Deblauwe et al. 2016; Title and Bemmels 2018). Moreover, WorldClim includes a broader and more globally consistent set of weather station inputs, which may reduce artifacts in areas with limited topographic complexity (Hijmans et al. 2005). WorldClim offers greater consistency with previous ecological niche modeling efforts in the Irano‐Turanian region, facilitating cross‐study comparisons. It is widely used in species distribution modeling due to its extensive climatic coverage, high spatial resolution, and compatibility with tools such as MaxEnt and ENMTools (Title and Bemmels 2018; Zurell et al. 2020), making it a robust and reliable choice for this study. We removed variables mean temperature of wettest quarter (bio8), mean temperature of driest quarter (bio9), precipitation of warmest quarter (bio18), and precipitation of coldest quarter (bio19) from all of the analyses because of known spatial artifacts (Escobar et al. 2014). Two‐step procedures were used to select subsets of the remaining 15 bioclimatic variables. First, we used Pearson correlation coefficients among the variables across the calibration area using R 3.5.0 software. One of each pair of variables was removed with a correlation ≥ 0.8 based on our knowledge of variable importance in the ecological conditions and habitat of the species. We applied the jackknife approach to select six sets of variables by removing the variable with the lowest independent contributions, creating sets of Mean Diurnal Range (bio2), Isothermality (bio3), Temperature Seasonality (bio4), Mean Temperature of Warmest Quarter (bio10), Mean Temperature of Coldest Quarter (bio11), Precipitation of Driest Month (bio14), Precipitation Seasonality (bio15), Precipitation of Wettest Quarter (bio16), and Precipitation of Driest Quarter (bio17) variables for analysis.

For future climate conditions, we downloaded climatic variables under future climate scenarios (SSP2‐4.5 and SSP5‐8.5) to match the present‐day datasets from the WorldClim website (https://www.worldclim.org/data/cmip6/cmip6_clim30s.html) at a spatial resolution of 30″. Four general circulation models (GCMs) were obtained for each scenario, including (1) Beijing Climate Center Climate System Model (BCC‐CSM2‐MR), (2) Australian Community Climate and Earth System Simulator Climate Model Version 2 (CCESS‐CM2), (3) Institute Pierre‐Simon Laplace‐Coupled Model Intercomparison Project (IPSL‐CM6A‐LR), and (4) Model for Interdisciplinary Research on Climate Version 6 (MIROC6) (Table S2). We used this diverse set of GCMs to illuminate the uncertainty in predictions of the potential distribution of the species in the future (Peterson et al. 2018).

### 2.3 Ecological Niche Models

We employed a simulation‐based approach to estimate a biologically realistic calibration region, referred to as M (following Barve et al. 2011), which represents the geographic area that has likely been accessible to the species through natural dispersal over relevant temporal scales. Accurate delineation of M is a critical step in ecological niche modeling, as it constrains background selection to ecologically meaningful areas within the species' potential dispersal range. This area forms the basis for background selection in ecological niche modeling and helps limit model calibration to ecologically meaningful regions, reducing bias and improving predictive reliability (Peterson et al. 2011). To delineate M, we considered the species' known distribution and modeled potential dispersal across the landscape under both constant (current) climate and glacial–interglacial climate change scenarios. These simulations incorporated processes of dispersal, colonization, and extinction using the Grinnell package in R (Barve et al. 2011; Machado‐Stredel et al. 2021; Amaro et al. 2023). The delineated M area encompasses regions that are both climatically and geographically accessible to the species, based on its documented distribution and inferred dispersal capacity. This delimitation explicitly excludes environmentally suitable but historically inaccessible areas, thereby ensuring ecological and biogeographical relevance. A detailed spatial representation of the estimated M region is presented in Figure 1b. We kept the default values for this analysis, except kernel spread, which we varied between 1 and 10; the simulation period was set to 100. Climatic data were masked to the hypothesized **M** area and a broader area of the Irano‐Turanian region for current and future distributions, respectively, using raster extraction routines in ArcGIS 10.3.1 (Figure 1b).

Ecological niche models were created using a maximum entropy algorithm for predicting the distribution of *T. dubia* across the Irano‐Turanian region. Despite advocacy for exploring various algorithms for fitting ecological niche models (Araújo 2007; Qiao et al. 2015), we chose to use only Maxent due to the differing probability types estimated by each algorithm, opting instead for a thorough exploration of Maxent's parameter space (Phillips et al. 2006; Peterson et al. 2011; Cobos et al. 2019). Maxent has also been shown to perform well with small sample sizes, producing robust and reliable predictions when model settings are carefully tuned (Hernandez et al. 2006; Wisz et al. 2008; van Proosdij et al. 2016), making it particularly suitable for studies involving narrowly distributed or data‐deficient species. We use the model‐selection procedure (Warren and Seifert 2011) via the kuenm package in R (Cobos et al. 2019). We explored all 29 possible combinations of the feature types (linear = l, quadratic = q, product = *p*, threshold = t, and hinge = h), and 12 regularization multiplier settings (0.1, 0.3, 0.5, 0.7, 0.9, 1, 1.25, 1.5, 1.75, 2, 3, and 4) and 6 combinations of the nine environmental variables. In all, 2088 candidate models were built using all possible combinations of these parameter values. We evaluated candidate models and selected the best models using the Akaike Information Criterion corrected for small sample sizes (Warren and Seifert 2011), performed significance tests using partial ROC (Peterson et al. 2008), and evaluated performance using a 5% training presence threshold to evaluate omission (Peterson et al. 2011). In more detail, candidate models were evaluated using partial ROC tests applied to 500 random replicate samples of 50% of the occurrences left out of model calibration (Peterson et al. 2008), and then statistical significance was evaluated via a direct count of replicates with AUC ratios ≤ 1.0. The threshold was done on all models based on an acceptable calibration omission rate (Peterson et al. 2008) of *E* = 5% and removed the models with omission rates above 0.05. Finally, models were filtered to retain only models with the lowest values of the Akaike Information Criterion (AICc) (Warren and Seifert 2011), retaining models with AICc values within one unit of the minimum.

We created final models using the parameter settings selected, performing 10 bootstrap replicates, the “logistic” output, and 10,000 background points in the kuenm package. The final model was transferred across a broad area of the Irano‐Turanian region under present‐day conditions for each of the eight future climate datasets (Wenger and Olden 2012; Yates et al. 2018), using all three model transfer options: free extrapolation (E), extrapolation and clamping (EC), and no extrapolation (NE).

We applied the median values across replicates as the best final estimate of suitability across the region for present‐day conditions. For projections to future conditions, we calculated the median of replicate medians across all 4 GCMs for SSP2‐4.5 and SSP5‐8.5. To generate binary maps for each of the present and future time periods, the replicates of final model outputs were thresholded to binary based upon an omission error criterion of *E* = 5% (Peterson et al. 2007), which allows a small proportion of known occurrences to be excluded to reduce overprediction and is widely applied when working with presence‐only data (Anderson et al. 2003; Pearson et al. 2007). The agreement of changes among the eight GCMs SSP2‐4.5 and SSP5‐8.5 scenarios was also used to represent changes in suitable areas in geographic projection. Specifically, we compared all projections to future conditions based on distinct GCMs against the current projection for each SSP scenario (Campbell et al. 2015). All analyses were performed in the kuenm R package (R Core Team 2018; Cobos et al. 2019) and ArcGIS 10.3.1. We also gathered a summary of the geographic locations and extent of protected areas in the Irano‐Turanian region, which encompasses the entire known distribution of the species, using data from the World Database on Protected Areas available at www.protectedplanet.net (Figure 5).

## 3 Results

We collected an initial dataset of 163 occurrence points for *T. dubia* across the study area. Eighty‐two records were omitted from the 163 original datasets based on the 1.5 km filtering distance and other data‐quality considerations. In total, the occurrence data comprised 81 unique occurrences for calibration and evaluation of ecological niche models (Table S2). Accessible areas are simulated under both changing frameworks of climatic conditions, including most of eastern Iran and small parts of Afghanistan and Pakistan. Figure 1 shows the georeferenced occurrence locations used in this study and the areas identified by our simulations to be accessible to *T. dubia* over time (M; the set of areas accessible to the species over relevant periods of its history) (Figure 1a,b).

Six climate variables were removed from the analyses based on high correlations (*r* > 0.8) and consequent collinearity among climatic variables. The remaining nine variables were retained in the jackknife analysis, including mean diurnal range (bio2), isothermality (bio3), temperature seasonality (bio4), mean temperature of the warmest quarter (bio10), mean temperature of the coldest quarter (bio11), precipitation of the driest month (bio14), precipitation seasonality (bio15), precipitation of the wettest quarter (bio16), and precipitation of the driest quarter (bio17). In six jackknife steps, bioclimatic variables bio3, bio4, bio14, bio15, and bio17 showed low contributions to model gain. Accordingly, six sets of bioclimatic variables were explored, including Set 1 (bio2‐bio3‐bio4‐bio10‐bio11‐bio14‐bio15‐bio16‐bio17); Set 2 (bio2‐bio3‐bio4‐bio10‐bio11‐bio14‐bio15‐bio16); Set 3 (bio2‐bio3‐bio4‐bio10‐bio11‐bio15‐bio16); Set 4 (bio2‐bio3‐bio4‐bio10‐bio11‐bio16); Set 5 (bio2‐bio3‐bio10‐bio11‐bio16); Set6 (bio2‐bio10‐bio11‐bio16). The influential bioclimatic variables in this study included mean diurnal range (bio2), isothermality (bio3), temperature seasonality (bio4), mean temperature of the warmest quarter (bio10), mean temperature of the coldest quarter (bio11), precipitation of the driest month (bio14), precipitation seasonality (bio15), precipitation of the wettest quarter (bio16), and precipitation of the driest quarter (bio17). Bio 16 was the most important variable, with 36.9% of the total variable contribution, followed by bio10, bio4, bio14, and bio11 with 23.5%, 9.5%, 6.9%, and 5.8% of the variable contribution, respectively (Figure S2; Table S3). The mean temperature of the warmest quarter (bio10) and precipitation of the wettest quarter (bio16) were the most important explanatory climatic variables based on nine variables. Based on the response curve of *T. dubia* to both climate variables (Figure S2), the precipitation of the wettest quarter (bio16) showed a peak between 60 and 70 mm, and habitat suitability sharply decreased in areas where the variable was less than 60 mm. Whereas the suitable mean temperature of the warmest quarter (bio10) of species ranged from 12°C to 37°C, with a peak at ~30°C, it was positively correlated with precipitation of the warmest quarter.

We assessed a total of 2088 candidate models for *T. dubia* using combinations of 29 feature classes, 12 regularization multipliers, and 6 environmental datasets. A total of 2055 models were statistically significantly better than random expectations based on the partial ROC test (*p* < 0.001; see Table S4). Of these models, 159 met the omission rate (OR) criteria (i.e., OR ≤ 0.15), and only one model was selected as the best model based on AICc. The best model included three feature types (linear, quadratic, and hinge), with one regularization parameter of 1.25 and nine climate variables in Set 1 (bio2‐bio3‐bio4‐bio10‐bio11‐bio14‐bio15‐bio16‐bio17).

**FIGURE 2:**
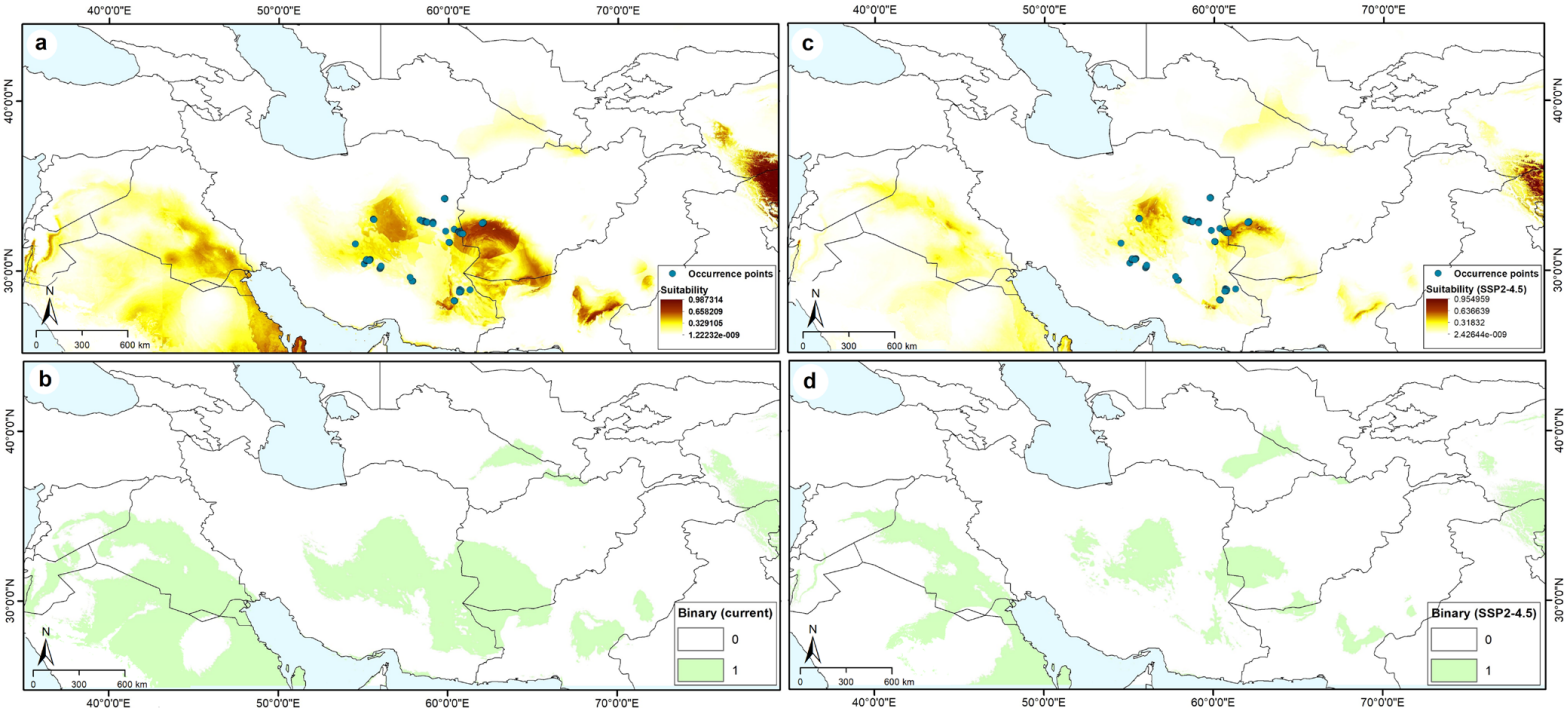
Current and future suitable areas for *Tamarix dubia* distribution based on Maxent model outputs across the study area. (a) Predicted areas of high suitability for present‐day conditions (median prediction). (b) The binary map under current based on a least training presence thresholding approach. (c) Predicted areas of high suitability for future conditions under scenarios of Shared Socioeconomic Pathways (SSP2‐4.5) across the study area. (d) The binary map under SSP2‐4.5 scenario based on a least training presence thresholding approach.

We found relatively broad current potential distributions of climate variables for *T. dubia* within central parts of the Irano‐Turanian region, despite its currently limited geographic distribution, suggesting some ecological flexibility within its core habitat. The potential distribution under current conditions for *T. dubia* included areas of high suitability across parts of Iran, Afghanistan, and a small part of Turkmenistan (IT region), Pakistan, Iraq, Saudi Arabia, Syria, Jordan, and Israel (Sahara–Sindian region) as well as China, India (CAR or Central‐Asiatic Region) (see Figure 2a). In the IT region, areas of highest suitability extended across the center and southeast of Iran, whereas suitability was markedly lower in the south (low elevation, with warm climatic conditions) and west and north (montane areas with cold climatic conditions) parts of Iran. In Iran, where *T. dubia* is most prevalent, our models predicted losses in parts of Kerman, Razavi Khorasan, and South Khorasan, while high suitability was identified only in small areas outside its existing range in Yazd, Sistan and Baluchestan, and a limited region of eastern South Khorasan. In contrast, the models forecast a broader area of high suitability in the southwestern provinces of Afghanistan (Figure S1a). Suitable areas were also identified in western Afghanistan and a small part of eastern Turkmenistan with low elevation (Figure 2a,b). Other suitable areas for the species were predicted in Sahara–Sindian and Central‐Asiatic regions.

**FIGURE 3:**
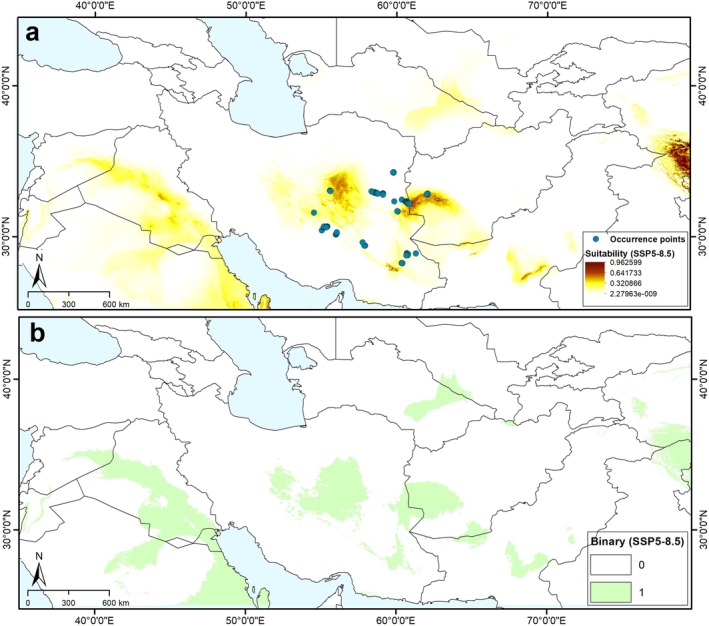
Future suitable areas and associated binary map for *Tamarix dubia* distribution based on Maxent model outputs under scenarios of Shared Socioeconomic Pathways (SSP5‐8.5) across the study area. (a) Predicted areas of high suitability for future conditions (median prediction). (b) Binary map based on a least training presence thresholding approach.

**FIGURE 4:**
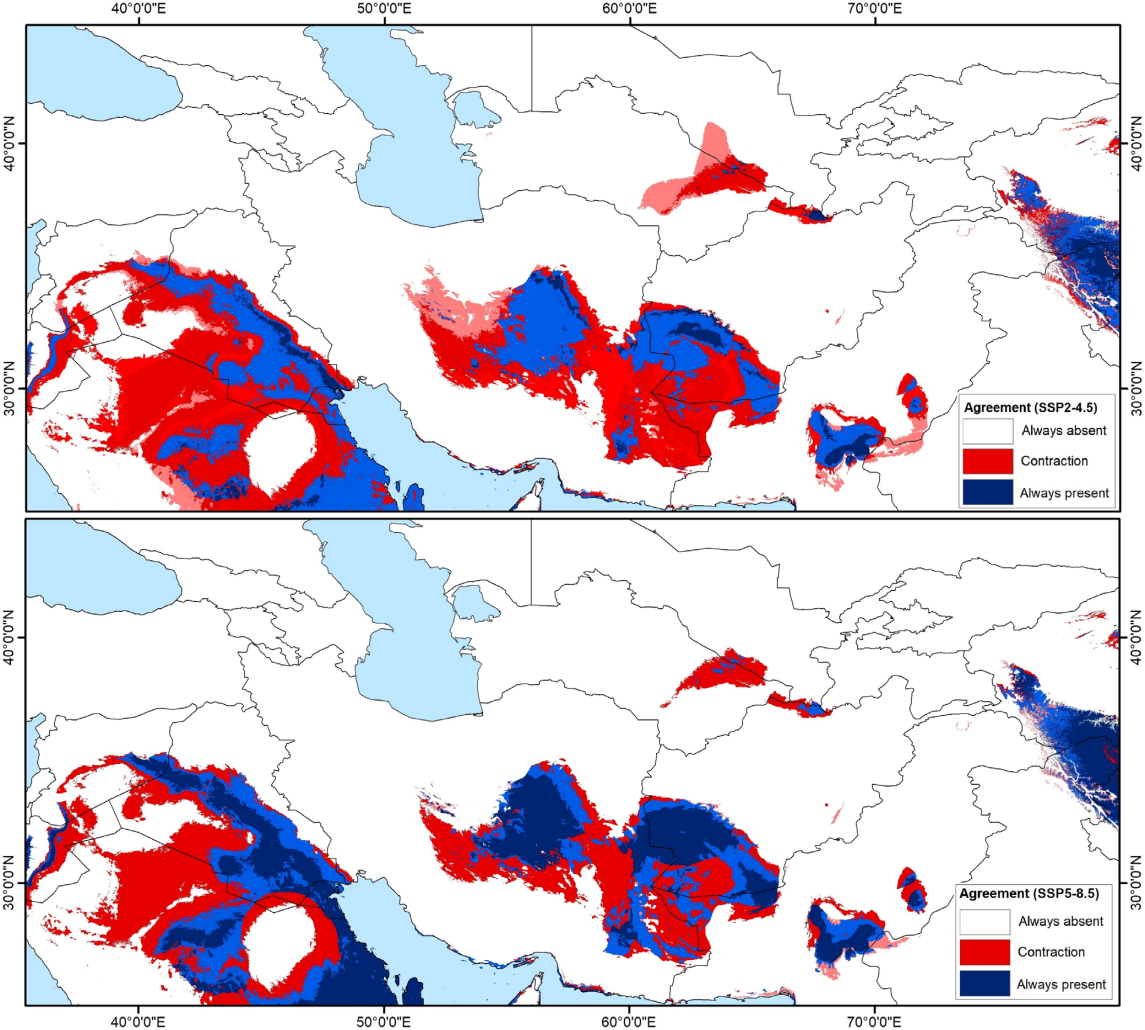
Predicted suitable areas and changes in suitability of *Tamarix dubia* based on Maxent model outputs under two climate change scenarios SSP2‐4.5 and SSP5‐8.5 and agreement between different general circulation models for the study area.

Model transfers to future climate scenarios revealed an impressive reduction under both SSP2‐4.5 and SSP5‐8.5 scenarios. Based on these scenarios, highly suitable areas expanded generally in a small part of central Iran, western Afghanistan (IT region), and a small part of western China and India (CAR region) (Figures 2c,d and 3a,b). Based on conditions anticipated for RCP 4.5 and RCP 8.5 (Figure 4), a decline of suitable areas was indicated in the areas of sub‐region IT2 in central Iran, western Afghanistan, a small part of Turkmenistan, and broad areas in the Sahara–Sindian region. In general, the models identified a future potential distribution that reflected the contraction of the distributional potential of *T. dubia* from the present distribution (Figure 4). Accordingly, the potential distribution of *T. dubia* decreased by 9.8% (842,970 km^2^) and 12.0% (1,019,128 km^2^) from current conditions to SSP2‐4.5 and SSP5‐8.5 conditions, respectively. Furthermore, the distributional area of the species decreased across the model calibration area (**M** area) by only 5.7% (380,128 km^2^) from current conditions to SSP2‐4.5 conditions and 6.0% (407,193 km^2^) to SSP5‐8.5 conditions (Table 1). Under both future scenarios, the suitable habitat range was predicted to decrease with global warming, which suggested *T. dubia* could not benefit from the changes in climate. A continuous temperature rise might have a negative effect on the geographical distribution of *T. dubia* , such that losses in suitable areas cause local extinction of the species in the future. These losses can also be observed on more local and more clearly accessible scales in actual ranges of the species under future climate scenarios (Figures 2, 3, 4 and S1b).

**TABLE 1:** Details of assessment of area (km^2^) and percentage of the potential distribution of *T. dubia* in the suitable and unsuitable areas for in Irano‐Turanian region and M area.

	Irano‐Turanian Region		M area	
	Area (Km^2^)	Percentage of the potential distribution	Area (Km^2^)	Percentage of the potential distribution
Suitable area				
Current	1,654,247	19.32%	613,109.4	8.58%
SSP2‐4.5	811,276.60	9.47%	232,981.8	2.93%
SSP5‐8.5	635,118.70	7.06%	205,916.6	2.53%
Unsuitable area				
Current	6,904,735	—	6,528,136	—
SSP2‐4.5	7,747,709	—	7,716,107	—
SSP5‐8.5	7,923,862	—	7,902,548	—
Contraction				
SSP2‐4.5	842,970.40	9.84%	380,127.6	5.65%
SSP5‐8.5	1,019,128	12.00%	407,192.8	6.04%

## 4 Discussion

Here, we projected the potential geographical distribution of *T. dubia* in the Irano‐Turanian region under both current and future climate scenarios and highlighted potential areas for the species. Our study found that the distribution of *T. dubia* was mainly controlled by four precipitation‐related bioclimatic variables (bio14: precipitation of driest month; bio15: precipitation seasonality; bio16: precipitation of the wettest quarter; bio17: precipitation of the driest quarter), five temperature‐related bioclimatic variables (bio2: mean diurnal range; bio3: Isothermality, bio4: temperature seasonality; bio10: mean temperature of the warmest quarter; bio11: mean temperature of the coldest quarter). Changes in precipitation and temperature patterns due to climate change can affect a wide range of physiological and ecological processes in plant species. For *T. buba* and related taxa, typically distributed in the warm‐temperate zones, such changes may disrupt water‐use efficiency, photosynthetic rates, stomatal conductance, and phenological timing, particularly under drought stress or elevated temperatures (Tognetti et al. 2007; Pol et al. 2010; Brant and Chen 2015). These species are especially sensitive to alterations in seasonal rainfall and heat stress, which can influence their survival, reproduction, and competitive dynamics.

Ecological niche models (ENMs) are powerful tools for identifying the climatic factors that drive species distributions and for guiding conservation strategies under rapidly changing environmental conditions (Elith and Leathwick 2009; Franklin 2010). In regions characterized by complex climatic heterogeneity, such as the Irano‐Turanian zone, the integration of biogeographically relevant classification systems enhances the ecological interpretation of model outputs. The global bioclimatic classification system proposed by Djamali et al. (2011) provides a region‐specific framework that links vegetation patterns with key climatic parameters, offering an ecologically meaningful context for species like *T. dubia* . According to this classification, *T. dubia* occupies six distinct bioclimatic zones that vary primarily in precipitation regimes and temperature extremes: (1) Mediterranean desertic‐continental (Mdc), characterized by low annual precipitation and prolonged summer drought; (2) Mediterranean xeric‐continental (Mxc), marked by summer drought and limited annual rainfall; (3) Mediterranean xeric‐oceanic (Mxo), featuring extended summer drought, low precipitation, and elevated winter temperature minima; (4) Tropical desertic (Trd), with a peak of summer rainfall in eastern Iran during July; (5) Mediterranean desertic‐oceanic (Mdo), characterized by longer summer drought and low annual precipitation; and (6) Tropical hyperdesertic (Trhd), defined by near year‐round drought and extreme summer heat (Djamali et al. 2011). Our modeling results align closely with this classification, identifying precipitation of the driest quarter (bio17), mean temperature of the warmest quarter (bio10), and mean temperature of the coldest quarter (bio11) as key predictors of the species' potential distribution. These findings underscore the critical role of climatic variables in shaping species presence in arid and semi‐arid ecosystems and demonstrate how bioclimatic stratification enhances the ecological relevance and interpretability of niche models, particularly in data‐limited regions.

The response curve demonstrates the relationship between environmental variables and the possible distribution of target species (also known as habitat suitability) and biological tolerances for the species and habitat preferences (Gebrewahid et al. 2020). The identification of the mean temperature of the warmest quarter (bio10) and precipitation of the wettest quarter (bio16) as the primary climatic predictors of *T. dubia* 's potential distribution highlights the species' sensitivity to thermal and moisture availability during critical growth periods. In arid and semi‐arid regions, plant species often exhibit narrow climatic tolerances, particularly to temperature extremes and water availability during seasonal transitions (Chesson et al. 2004; Schwinning and Sala 2004). Temperature plays a central role in regulating phenological events such as germination, flowering, and seed maturation, while episodic precipitation pulses often drive growth and reproductive success in xerophytic species (Noy‐Meir 1973). The significance of bio16 suggests that *T. dubia* may rely on sufficient wet‐season rainfall for key physiological processes, including nutrient uptake and cellular hydration, which are constrained in lower precipitation environments. Similarly, the importance of bio10 may reflect the species' thermal optimum for photosynthesis and enzyme activity, aligning with previous findings that warm‐season temperatures critically influence the metabolic performance and range limits of many temperate and desert‐adapted taxa (O'Brien et al. 2016; Dai et al. 2019). These results underscore the ecological importance of seasonal climatic extremes in shaping species distributions and suggest that future shifts in these variables due to climate change may disproportionately impact *T. dubia* 's range and habitat suitability.

Our results indicate that, despite its limited known distribution, *T. dubia* currently occupies climatically suitable habitats across parts of the central Irano‐Turanian region, pointing to an ecological potential that extends beyond its observed range. Ecological niche modeling suggests that *T. dubia* may experience both regional contractions and expansions in suitable habitat under present and projected future climate scenarios. While newly suitable areas are projected outside the species' current distribution, particularly in portions of the Sahara–Sindian and Central‐Asiatic regions, it remains uncertain whether *T. dubia* possesses the dispersal capacity and ecological adaptability necessary to colonize and persist in these climatically favorable but geographically distant habitats. The ability of the species to access and successfully colonize these disjunct areas will depend on its dispersal capabilities. Previous studies on the related species *Tamarix indica* indicate that *Tamarix* species produce massive numbers of seeds each year (Glenn and Nagler 2005). It seems that massive numbers of seeds can be the main propagule for the dispersal of the species. Moreover, a symmetrical radial pappus on one end of the seed might function as an accessory structure that provides a buoyancy force and promotes long‐distance seed dispersal by facilitating seed dispersal through the air or on water (Merkel and Hopkins 1957). They are also able to produce roots from broken‐off stems and branches, which can sprout in sediment (Natale et al. 2010). Although these mechanisms could aid in dispersal, it is unlikely that seeds can move several thousand kilometers to the Sahara–Sindian and Central‐Asiatic regions. Even if *Tamarix* seeds reach areas close to their actual ranges, they would encounter various types of stress for colonization, including drought, high soil salinity, high temperatures, and strong light conditions due to the large deficit between precipitation and potential evapotranspiration in desert areas (Natale et al. 2010). Therefore, based on these mechanisms and regardless of habitat suitability, it seems unlikely that *T. dubia* would be able to colonize the most distant areas.

Our models indicate a notable reduction in climatically suitable habitat for *T. dubia* under both moderate (SSP2‐4.5) and high (SSP5‐8.5) future climate scenarios. Although the projected losses across the broader calibration region are moderate, the declines within the species' more accessible and currently occupied habitats suggest potential range contraction and increased vulnerability to local extinction. These findings are consistent with broader ecological patterns, where endemic and range‐restricted species in arid and semi‐arid regions are especially susceptible to the compounded effects of warming and reduced precipitation (Engler et al. 2011; Pacifici et al. 2015). Unlike some generalist taxa that may track shifting climate envelopes, narrowly adapted species like *T. dubia* may lack the ecological flexibility or dispersal capacity to colonize new areas, particularly under rapid climate change scenarios. Furthermore, climate‐driven habitat loss may exacerbate population fragmentation and reduce genetic diversity, increasing extinction risk (Opdam and Wascher 2004). These results emphasize the importance of incorporating climate vulnerability assessments into regional conservation planning for endemic flora in the Irano‐Turanian zone.

In Iran, significant losses in the suitable areas of *T. dubia* under future conditions were predicted in four provinces: Sistan and Baluchestan, Kerman, South Khorasan, and Razavi Khorasan (Figure S1b). These losses are particularly concerning, as gene dispersal routes for *T. dubia* have been suggested to extend from Sistan and Baluchestan to South Khorasan (Figure S3) (Ijbari et al. 2024). Ijbari et al. (2024) found that genetic variability is lower within *T. dubia* populations than among populations across Iran, noting that genetic distance between populations increases with geographical separation, which correlates with reduced gene flow. Furthermore, the limited genetic diversity within populations may result from intrapopulation pollination, exacerbated by the significant distances between neighboring populations. The restricted number of trees in each area also contributes to genetic drift and a decline in genetic diversity (Ellstrand and Elam 1993; Frankham et al. 2002). They identified environmental factors and geographic distance as primary reasons for the minimal gene flow among *T. dubia* populations (Hamrick and Godt 1996; Jump and Peñuelas 2005). Consequently, low genetic diversity within these populations can hinder their ability to adapt to changing environmental conditions (Reed and Frankham 2003). This adaptability is crucial for enabling populations to respond to new challenges, such as diseases or climate change, as it increases the likelihood of possessing advantageous traits (Spielman et al. 2004; Hoffmann and Sgrò 2011). Populations with limited genetic diversity may lack the necessary variation to survive fluctuations in their habitats, making them more vulnerable to extinction over time (Li 2018a, 2018b; Frankham 2005).

Moreover, recent studies have emphasized the value of integrating ecological niche modeling (ENM) with population genetic analyses to provide a more comprehensive understanding of species' responses to environmental change. Such integrative approaches can reveal historical processes, identify dispersal barriers, and assess the potential for future adaptation. For example, Carnaval et al. (2009) demonstrated that regions of long‐term climatic stability correspond with areas of high genetic diversity in the Brazilian Atlantic Forest, highlighting the importance of refugia in maintaining evolutionary potential. Similarly, Knowles et al. (2007) combined ecological niche models with multilocus genetic data to reconstruct past population dynamics and range shifts in montane insects, showing how niche stability influenced genetic structure. In the Mediterranean region, Ortego et al. (2012) used both SDM and AFLP markers to reveal that habitat fragmentation significantly reduced gene flow and increased genetic differentiation in *Juniperus thurifera*, a drought‐tolerant conifer. These studies reinforce our findings that *Tamarix dubia*, which exhibits low genetic diversity (Ijbari et al. 2024) and limited dispersal potential, may face substantial constraints in adapting to and colonizing newly suitable areas projected under future climate scenarios. Thus, the integration of genetic data with spatial models offers critical insights into the vulnerability and long‐term persistence of endemic species like *T. dubia* in arid and semi‐arid regions.

Notably, the wide areas of Yazd and Farah provinces as well as small parts of Sistan and Baluchestan, and South Khorasan were anticipated as highly suitable, which could be important for conservation programs if the species can move to these areas. Our results are contrary to what has been found for other species in this region (Behroozian et al. 2020; Karami et al. 2022; Noedoost et al. 2024). Previous findings reported an increase in the extension of the suitable areas of endemic plant species such as *Dianthus polylepis* (Behroozian et al. 2020), *Nepeta glomerulosa* (Karami et al. 2022), and *Achillea eriophora* (Noedoost et al. 2024) in their future ranges. However, soil salinization and aridification of the species' ranges under global warming can strengthen the losses in suitable areas of the species (Zhang et al. 2018; Sun et al. 2020). Moreover, the habitat suitability of *T. dubia* can be affected by several essential factors such as environmental factors, intraspecific facilitation, wind dispersal of seeds, and human activities. The intensive dispersal and germination in the short term as well as unsustainable land use and management lead to habitat fragmentation and a decrease in the suitable habitat of *T. dubia* (Jiao et al. 2021; Wu et al. 2022). Furthermore, a recent study utilized ENM alongside field surveys to improve conservation management strategies for a rare plant species in Iran, *Dianthus pseudocrinitus* Behrooz. & Joharchi, highlighting the role of ENM in identifying key areas and predicting species' distributions, which in turn are essential for effective conservation planning (Behroozian et al. 2022).

In total, the substantial reduction in climatically suitable habitat projected for *T. dubia* under both future climate scenarios is one of the most critical findings of this study. This outcome is particularly concerning given the species' already narrow ecological niche and fragmented distribution across the Irano‐Turanian region. Species with limited geographic ranges and specific environmental requirements often exhibit low adaptive capacity and heightened sensitivity to climatic shifts (Thuiller et al. 2009; Urban 2015). In this respect, our results are consistent with global assessments indicating that endemic and range‐restricted taxa, especially in arid and semi‐arid biomes, are disproportionately vulnerable to climate change (Warren et al. 2013; Trabucco et al. 2021). Furthermore, the predicted loss of suitable habitat in provinces such as Sistan and Baluchestan and Kerman is ecologically significant, as these areas serve as refugia for numerous stress‐tolerant plant species and support unique assemblages within desert–steppe ecosystems. These findings underscore the urgent need to integrate climate vulnerability assessments into regional conservation strategies—not only for *T. dubia* but for a broader suite of endemic taxa facing similar environmental constraints.

## 5 Caveats

It is essential to acknowledge the limitations inherent in our modeling framework. While our results closely align with the known distribution of *T. dubia* , several methodological and ecological considerations warrant caution in interpreting the biological and geographic implications of our findings. Correlative niche models, such as MaxEnt, are subject to common challenges, most notably the limited availability and spatial bias of occurrence data. Insufficient or unevenly distributed records can result in inaccurate representations of a species' ecological niche and potential distribution (Stockwell and Peterson 2002; Wisz et al. 2008). To mitigate this, we employed robust methodologies using Maxent, supplemented by jackknife and model‐selection approaches for model tuning (Phillips et al. 2006; Shcheglovitova and Anderson 2013; Aiello‐Lammens et al. 2015). Additionally, we addressed biases in geographic sampling by thinning occurrence data and removing clumped or artificially clustered records (Boria et al. 2014). While Maxent remains a widely used and effective tool for presence‐only modeling, future work could benefit from incorporating ensemble modeling approaches or alternative algorithms to improve prediction accuracy and account for methodological uncertainty (e.g., Thuiller et al. 2009; Hao et al. 2019).

A crucial yet often underexplored aspect is whether the distribution of *T. dubia* corresponds with its environmental conditions and available landscapes. Biotic interactions, dispersal limitations, and anthropogenic activities can disrupt this estimate, complicating the quantification of real habitat use for these species. Consequently, niches estimated via these methods may not accurately capture the fundamental ecological constraints on distribution (Huntley et al. 2010). In our study, we employed Maxent as a correlative modeling approach to explore the potential distribution of *T. dubia* across a broad spatial scale. However, we urge caution in interpreting these results, as our models do not account for factors such as time‐lagged responses, historical processes, or complex biotic interactions. Future studies should consider incorporating such variables to improve inference and predictive capacity.

Finally, the suggestion that *T. dubia* may face an elevated extinction risk under future climatic scenarios should be regarded as preliminary. While our projections indicate a contraction in climatically suitable habitat and reveal multiple ecological vulnerabilities, formal conservation assessments, such as those required by IUCN Red List criteria, depend on comprehensive demographic data, including population size, reproductive rates, and long‐term trends (IUCN Standards and Petitions Committee 2022). Nevertheless, the combination of projected range loss, low genetic diversity, and limited dispersal capacity highlights a potential conservation concern for *T. dubia* . These findings emphasize the need for proactive monitoring and early management measures to address the impacts of climate‐induced habitat degradation.

## 6 Applications for Conservation

We investigated the distributional potential of *Tamarix dubia* under current and future climate conditions. This information is critical for the conservation of the species, as it underscores the significance of areas severely impacted by climate change. For instance, our models anticipate significant losses in many suitable areas under future scenarios. While some areas may remain suitable, most of the species' current ranges are expected to experience a contraction in suitability, indicating that conservation efforts should focus on these regions (Figure 4).

An important factor to consider in interpreting our climate‐based results is the prolonged and severe drought that has affected southeastern Iran, particularly in the provinces of Sistan and Baluchestan, Kerman, and South Khorasan, since the late 1990s and into the 2010s, characterized by significant declines in precipitation and increasing aridity (Raziei et al. 2014; Modarres and Sarhadi 2011). Although human activities do not directly impact *T. dubia* , climate change, characterized by reduced water availability, increased evaporation, strong winds, and compounded geoclimatic effects, has jeopardized its ranges. Moreover, recent political decisions to block Afghan rivers from entering regional basins have intensified drought conditions, with global warming likely to exacerbate these effects in the future (Ahmadi et al. 2023; Mohammadi 2024).

Given the regional impacts of climate change, including extreme water scarcity and the losses predicted by our models, *T. dubia* may indeed threaten or endanger. Particularly concerning are the regions within the species' current geographic distribution in Sistan and Baluchestan, Kerman, and South Khorasan, where our models anticipate substantial contractions in suitable areas. This geographic‐scale reduction in range could have significant implications for the status of *T. dubia* populations and their resilience to other adverse factors. This geographic‐scale reduction in range could lead to decreased genetic diversity, heightened population fragmentation, and increased extinction risk—patterns observed in other species experiencing climate‐driven range losses (Thomas et al. 2004; Pacifici et al. 2015; Bellard et al. 2012). For instance, in Mediterranean and alpine regions, several plant species have shown reduced reproductive success and local extirpations following habitat loss due to shifting climate envelopes (Engler et al. 2011; Dullinger et al. 2012). Similar outcomes could compromise the long‐term viability and resilience of *T. dubia* populations in their native habitats.

**FIGURE 5:**
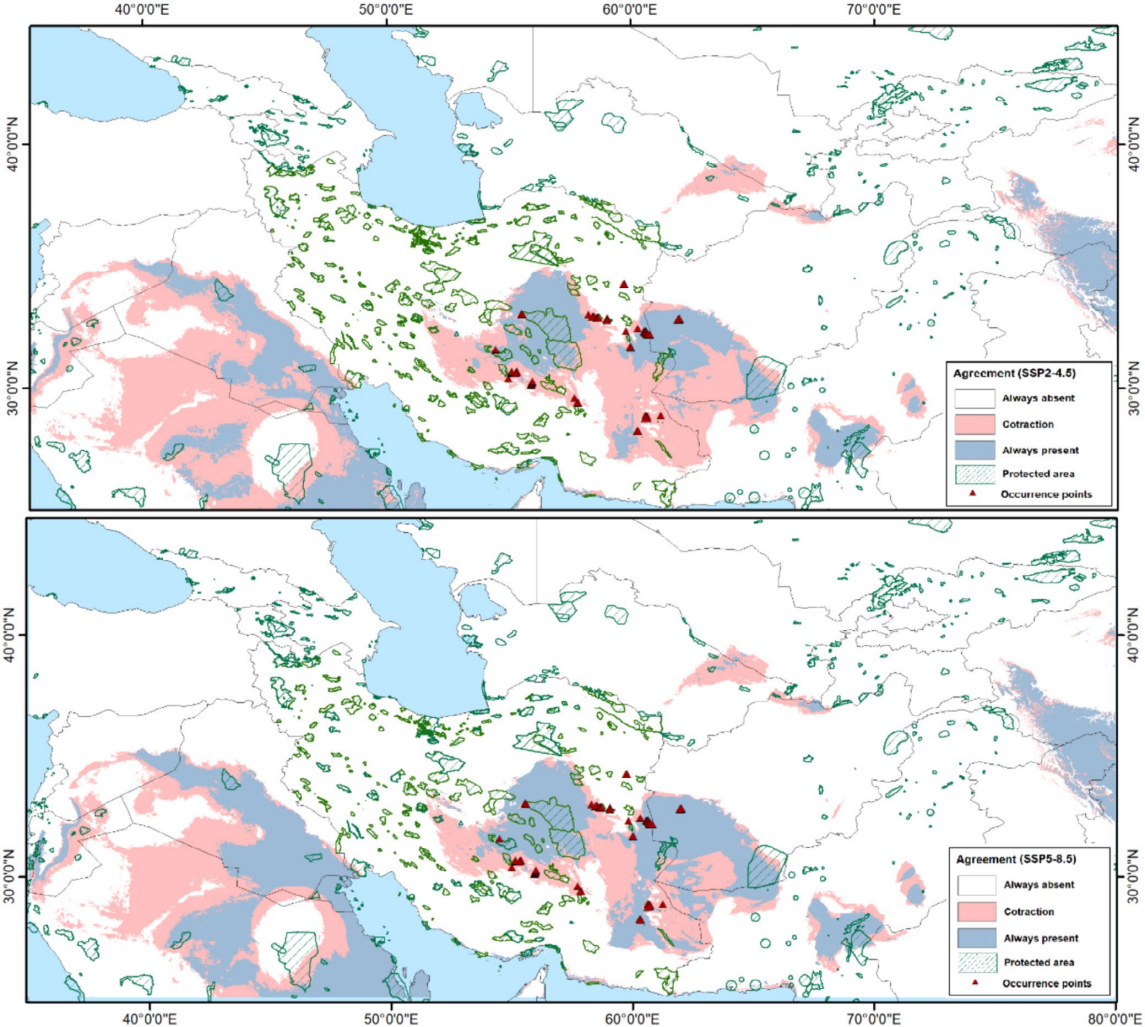
Location of Protected Areas in the Irano‐Turanian region. Habitat suitability and contraction areas for *Tamarix dubia* in relation to the position and extent of protected areas within the study area (given by www.protectedplanet.net).

On the other hand, high‐suitability areas in Yazd, Sistan and Baluchestan, South Khorasan, and Afghanistan should be prioritized for conservation planning, including the creation or expansion of protected areas. These regions represent a crucial first step in the effective conservation and scientific management of *Tamarix dubia*. Notably, significant portions of these areas currently lack protection in Iran, and a substantial expanse in Afghanistan is unprotected (Figure 5). This study provides valuable insights into the potential effects of climate change on *T. dubia* and supports the conservation and management of endemic species in arid and semi‐arid regions. It also lays the groundwork for future research aligned with conservation efforts. We recommend implementing conservation strategies consistent with our findings, including both in situ and *ex situ* conservation methods, conventional seed bank storage as well as other conservation approaches (e.g., Syfert et al. 2014; Sofaer et al. 2019; Eyre et al. 2022) for future studies. Furthermore, we suggest that future studies apply formal conservation assessments based on IUCN Red List criteria, incorporating metrics such as extent of occurrence, area of occupancy, population trends, and threats, to provide a rigorous evaluation of the conservation status of *T. dubia* .

## 7 Conclusion

The results of the present study provide useful findings for discussing the potential effect of climate change on the habitats of *T. dubia* , an endemic species in the Irano‐Turanian region. As a result, a decreased distribution pattern of *T. dubia* was presented using two climate change scenarios (SSP2‐4.5 and SSP5‐8.5). Furthermore, the environmental factors affecting the suitability of *T. dubia* were estimated, and the results indicated that the *T. dubia* species preferred a warm and moderately moist climate, with key environmental predictors including the mean temperature of the warmest quarter and precipitation of the wettest quarter. In addition to climate‐based habitat loss, our findings suggest that *T. dubia* faces compounding risks due to limited dispersal capacity and low genetic diversity, which could reduce its ability to colonize newly suitable areas under future climatic conditions. The integration of ecological niche modeling with insights from population genetics underscores the vulnerability of narrowly distributed endemic species like *T. dubia* to environmental change. These results emphasize the urgency of implementing targeted conservation strategies, including habitat management, monitoring, and potential ex situ conservation, to mitigate the risk of local extinction and support the resilience of *T. dubia* in an increasingly arid and fragmented landscape.

## Author Contributions

**Habibollah Ijbari:** conceptualization (equal), formal analysis (equal), methodology (equal), writing – review and editing (supporting). **Jamil Vaezi:** investigation (equal), project administration (equal), supervision (equal), writing – review and editing (equal). **Maryam Behroozian:** conceptualization (equal), formal analysis (equal), methodology (equal), validation (equal), writing – original draft (equal). **Hamid Ejtehadi:** conceptualization (equal), funding acquisition (equal), project administration (equal), supervision (equal), writing – review and editing (equal).

## Conflicts of Interest

The authors declare no conflicts of interest.

## Supporting information


Data S1.

Table S4.

## Data Availability

The data that supports the findings of this study are available in the Supporting Information of this article.
