# Supplementary material for: Impact of Climate Change on the Distributional Potential of the Endemic Species Tamarix dubia Bunge and Conservation Implications for the Irano‐Turanian Region
Source: Ecol Evol. 2025 Jul 28;15(8):e71877. doi: 10.1002/ece3.71877 (PMC12304089; doi:10.1002/ece3.71877)

Supplementary

**Impact of Climate Change on the Distributional Potential of the Endemic Species Tamarix dubia Bunge and Conservation Implications for the Irano-Turanian Region**

Habibollah Ijbari^1^, Jamil Vaezi^1^, Maryam Behroozian^2^, Hamid Ejtehadi^1,3^*

^1^Quantitative Plant Ecology and Biodiversity Research Lab, Department of Biology, Faculty of Science, Ferdowsi University of Mashhad, Mashhad, Iran

[*h.ijbari@yahoo.com*](mailto:h.ijbari@yahoo.com)*;* [*hejtehadi@um.ac.ir*](mailto:hejtehadi@um.ac.ir); [*vaezi@um.ac.ir*](mailto:vaezi@um.ac.ir)

^2^Herbarium FUMH, Ferdowsi University of Mashhad, Mashhad, Iran

*maryam.behroozian94@gmail.com*

^3^School of Environmental Sciences, University of Guelph, Guelph, Ontario, Canada

[*manand@uoguelph.ca*](mailto:manand@uoguelph.ca)*; ejtehadh@uoguelph.ca*

Corresponding author:

E–mail: *hejtehadi@um.ac.ir*

Address: Quantitative Plant Ecology and Biodiversity Research Lab, Department of Biology, Faculty of Science, Ferdowsi University of Mashhad, Mashhad, Iran

**Table S1.** Summary of occurrence data used in this study

| **Address** | **Province** | **Country** | **longitude** | **latitude** | **Elevation** |
| --- | --- | --- | --- | --- | --- |
| Mirjaveh, Khash, Ladiz | Sistan and Baluchestan | Iran | 61.2611412 | 28.86464 | 1332 amsl |
| Zahedan, Khash, Kamzard | Sistan and Baluchestan | Iran | 60.6448113 | 28.87763 | 1787 amsl |
| Zahedan, Khash, Kamzard | Sistan and Baluchestan | Iran | 60.6811699 | 28.85645 | 1848 amsl |
| Zahedan, Khash, Kamzard | Sistan and Baluchestan | Iran | 60.6998179 | 28.84059 | 1890 amsl |
| Zahedan, Khash, Kamzard | Sistan and Baluchestan | Iran | 60.7301576 | 28.83619 | 1942 amsl |
| Zahedan, Khash, Kamzard | Sistan and Baluchestan | Iran | 60.7208317 | 28.75814 | 1908 amsl |
| Zahedan, Khash, Kamzard | Sistan and Baluchestan | Iran | 60.6737681 | 28.75536 | 1832 amsl |
| Zahedan, Khash, Mirabad | Sistan and Baluchestan | Iran | 60.6221358 | 28.74256 | 1753 amsl |
| Nukabad, Bazman, Gohar Kuh | Sistan and Baluchestan | Iran | 60.34919 | 28.22475 | 1338 amsl |
| Nukabad, Bazman, Gohar Kuh | Sistan and Baluchestan | Iran | 60.3269023 | 28.23762 | 1223 amsl |
| Nukabad, Bazman, Gohar Kuh | Sistan and Baluchestan | Iran | 60.3087164 | 28.23723 | 1166 amsl |
| Nukabad, Bazman, Gohar Kuh | Sistan and Baluchestan | Iran | 60.3300385 | 28.21225 | 1253 amsl |
| Kerman, Bam | Kerman | Iran | 57.8768702 | 29.38524 | 1708 amsl |
| Kerman, Bam | Kerman | Iran | 57.8687991 | 29.39601 | 1722 amsl |
| Kerman, Bam | Kerman | Iran | 57.8334246 | 29.39527 | 1756 amsl |
| Kerman, Bam | Kerman | Iran | 57.720505 | 29.60007 | 2162 amsl |
| Rafsanjan, Mes-e Sarcheshmeh | Kerman | Iran | 55.9480637 | 30.1181 | 2225 amsl |
| Rafsanjan, Mes-e Sarcheshmeh | Kerman | Iran | 55.9621332 | 30.16156 | 2090 amsl |
| Rafsanjan, Mes-e Sarcheshmeh | Kerman | Iran | 55.9854418 | 30.19417 | 1961 amsl |
| Rafsanjan, Mes-e Sarcheshmeh | Kerman | Iran | 55.9892402 | 30.21503 | 1893 amsl |
| Rafsanjan, Mes-e Sarcheshmeh | Kerman | Iran | 56.0063843 | 30.26312 | 1712 amsl |
| Shahr-e Babak, Jowzam | Kerman | Iran | 55.009312 | 30.39344 | 2188 amsl |
| Jowzam, Anar | Kerman | Iran | 55.1376015 | 30.59935 | 1922 amsl |
| Jowzam, Anar | Kerman | Iran | 55.1399798 | 30.61508 | 1894 amsl |
| Jowzam, Anar | Kerman | Iran | 55.1413698 | 30.63484 | 1861 amsl |
| Jowzam, Anar | Kerman | Iran | 55.1578469 | 30.65977 | 1808 amsl |
| Anar, Bayaz | Kerman | Iran | 55.3786752 | 30.66895 | 1588 amsl |
| Anar, Bayaz | Kerman | Iran | 55.3503271 | 30.65524 | 1655 amsl |
| Anar, Bayaz | Kerman | Iran | 55.3400186 | 30.63903 | 1699 amsl |
| Anar, Bayaz | Kerman | Iran | 55.3301922 | 30.62184 | 1737 amsl |
| Anar, Bayaz | Kerman | Iran | 55.3086702 | 30.59832 | 1807 amsl |
| Mehriz road, Anar | Kerman | Iran | 54.484392 | 31.5681 | 1445 amsl |
| Yazd, Tabas | Yazd | Iran | 55.5548062 | 33.04414 | 1161 amsl |
| Yazd, Tabas | Yazd | Iran | 55.5719074 | 33.02833 | 1223 amsl |
| Yazd, Tabas | Yazd | Iran | 55.5889515 | 33.01428 | 1312 amsl |
| Deyhuk, Khusf, Tilkhour | South Khorasan | Iran | 58.2997245 | 32.99785 | 1254 amsl |
| Deyhuk, Khusf, Tilkhour | South Khorasan | Iran | 58.4929279 | 32.92114 | 1154 amsl |
| Deyhuk, Khusf, Tilkhour | South Khorasan | Iran | 58.5350508 | 32.94703 | 1207 amsl |
| Deyhuk, Khusf, Tilkhour | South Khorasan | Iran | 58.4886782 | 32.9083 | 1143 amsl |
| Deyhuk, Khusf, Tilkhour | South Khorasan | Iran | 58.4519132 | 32.90141 | 1113 amsl |
| Deyhuk, Khusf, Tilkhour | South Khorasan | Iran | 58.6257262 | 32.8638 | 1285 amsl |
| Deyhuk, Khusf, Tilkhour | South Khorasan | Iran | 58.6499813 | 32.86495 | 1321 amsl |
| Deyhuk, Khusf, Tilkhour | South Khorasan | Iran | 58.6716702 | 32.87521 | 1365 amsl |
| Deyhuk, Khusf, Tilkhour | South Khorasan | Iran | 58.6895816 | 32.85715 | 1404 amsl |
| Deyhuk, Khusf, Tilkhour | South Khorasan | Iran | 58.7069415 | 32.84599 | 1438 amsl |
| Deyhuk, Khusf, Tilkhour | South Khorasan | Iran | 58.7225882 | 32.85525 | 1492 amsl |
| Khusf, Birjand, Rakat | South Khorasan | Iran | 59.0715966 | 32.85394 | 1489 amsl |
| Khusf, Birjand, Rakat | South Khorasan | Iran | 59.0645813 | 32.83503 | 1566 amsl |
| Khusf, Birjand, Rakat | South Khorasan | Iran | 59.0614341 | 32.79391 | 1721 amsl |
| Khusf, Birjand, Rakat | South Khorasan | Iran | 59.0635984 | 32.7693 | 1824 amsl |
| Sarbisheh, Doreh | South Khorasan | Iran | 60.3180926 | 32.43025 | 1384 amsl |
| Doreh, Mahirud | South Khorasan | Iran | 60.5630389 | 32.31095 | 1249 amsl |
| Doreh, Mahirud | South Khorasan | Iran | 60.5830196 | 32.3052 | 1227 amsl |
| Doreh, Mahirud | South Khorasan | Iran | 60.6048794 | 32.30041 | 1198 amsl |
| Doreh, Mahirud | South Khorasan | Iran | 60.6246166 | 32.31085 | 1199 amsl |
| Doreh, Mahirud | South Khorasan | Iran | 60.6516939 | 32.33541 | 1306 amsl |
| Doreh, Mahirud | South Khorasan | Iran | 60.6320493 | 32.28727 | 1150 amsl |
| Doreh, Mahirud | South Khorasan | Iran | 60.6494089 | 32.26914 | 1107 amsl |
| Doreh, Mahirud | South Khorasan | Iran | 60.6241044 | 32.25819 | 1211 amsl |
| Doreh, Mahirud | South Khorasan | Iran | 60.6752395 | 32.27783 | 1142 amsl |
| Doreh, Mahirud | South Khorasan | Iran | 60.6743982 | 32.23334 | 1034 amsl |
| Doreh, Mahirud | South Khorasan | Iran | 60.6880802 | 32.21477 | 985 amsl |
| Doreh, Mahirud | South Khorasan | Iran | 60.6992762 | 32.19808 | 946 amsl |
| Doreh, Mahirud | South Khorasan | Iran | 60.72465 | 32.19468 | 985 amsl |
| Doreh, Mahirud | South Khorasan | Iran | 60.7106909 | 32.17008 | 907 amsl |
| Doreh, Mahirud | South Khorasan | Iran | 60.77351 | 32.18859 | 1093 amsl |
| Doreh, Mahirud | South Khorasan | Iran | 60.8275733 | 32.1694 | 939 amsl |
| Sarbisheh, Nehbandan | South Khorasan | Iran | 59.82659 | 32.31648 | 1610 amsl |
| Nehbandan, Shusef | South Khorasan | Iran | 60.0551568 | 31.65874 | 1428 amsl |
| Nehbandan, Shusef | South Khorasan | Iran | 60.030279 | 31.67189 | 1519 amsl |
| Nehbandan, Shusef | South Khorasan | Iran | 60.0095786 | 31.66745 | 1584 amsl |
| Jija Sarai | Farah | Afghanistan | 62.021022 | 32.79672 | 998 amsl |
| Jija Sarai | Farah | Afghanistan | 62.001276 | 32.80312 | 930 amsl |
| Jija Sarai | Farah | Afghanistan | 61.985153 | 32.80624 | 909 amsl |
| Jija Sarai | Farah | Afghanistan | 61.991097 | 32.78489 | 963 amsl |
| Jija Sarai | Farah | Afghanistan | 62.028297 | 32.82152 | 938 amsl |
| Jija Sarai | Farah | Afghanistan | 62.029784 | 32.84967 | 903 amsl |
| Jija Sarai | Farah | Afghanistan | 61.96871 | 32.79655 | 915 amsl |
| Khaf, Kabir Kuh | Razavi Khorasan | Iran | 59.771338 | 34.23685 | 1031 amsl |
| Khaf, Kabir Kuh | Razavi Khorasan | Iran | 59.791296 | 34.26096 | 970 amsl |
| Khaf, Kabir Kuh | Razavi Khorasan | Iran | 59.80627 | 34.24425 | 930 amsl |

**Table S2.** A list of models used for SSP2-4.5 and SSP2-8.5 scenarios.

| **No.** | **Model name** | **Abbreviation name** |
| --- | --- | --- |
| **1** | Beijing Climate Center (BCC) Climate System Model | BCC-CSM2-MR |
| **2** | Australian Community Climate and Earth System Simulator Climate Model Version 2 | CCESS-CM2 |
| **3** | Institut Pierre-Simon Laplace- Coupled Model Intercomparison Project | IPSL-CM6A-LR |
| **4** | Model for Interdisciplinary Research on Climate Version 6 | MIROC6 |

**Table S3.** Percentage contribution (PC) and permutation importance (PI) of the bioclimatic variables for *Dionysia diapensiifolia*

| Variable | Percent contribution (PC) | Permutation importance (PI) |
| --- | --- | --- |
| bio16 | 36.9 | 28.3 |
| bio10 | 23.5 | 2.6 |
| bio4 | 9.5 | 20.9 |
| bio14 | 6.9 | 3.4 |
| bio11 | 5.8 | 22.8 |
| bio3 | 5.6 | 2.2 |
| bio2 | 4.6 | 9.2 |
| bio15 | 4.1 | 6.8 |
| bio17 | 3 | 3.7 |

**Table S4.** Summary of model parameter settings explored and tested in this study for *Tamarix dubia.*

(Excel file)

**Figure S1.** Present-day (a) and future (b) suitable areas for *Tamarix dubia* distribution as well as occurrence points in the provinces of Iran and Afghanistan.


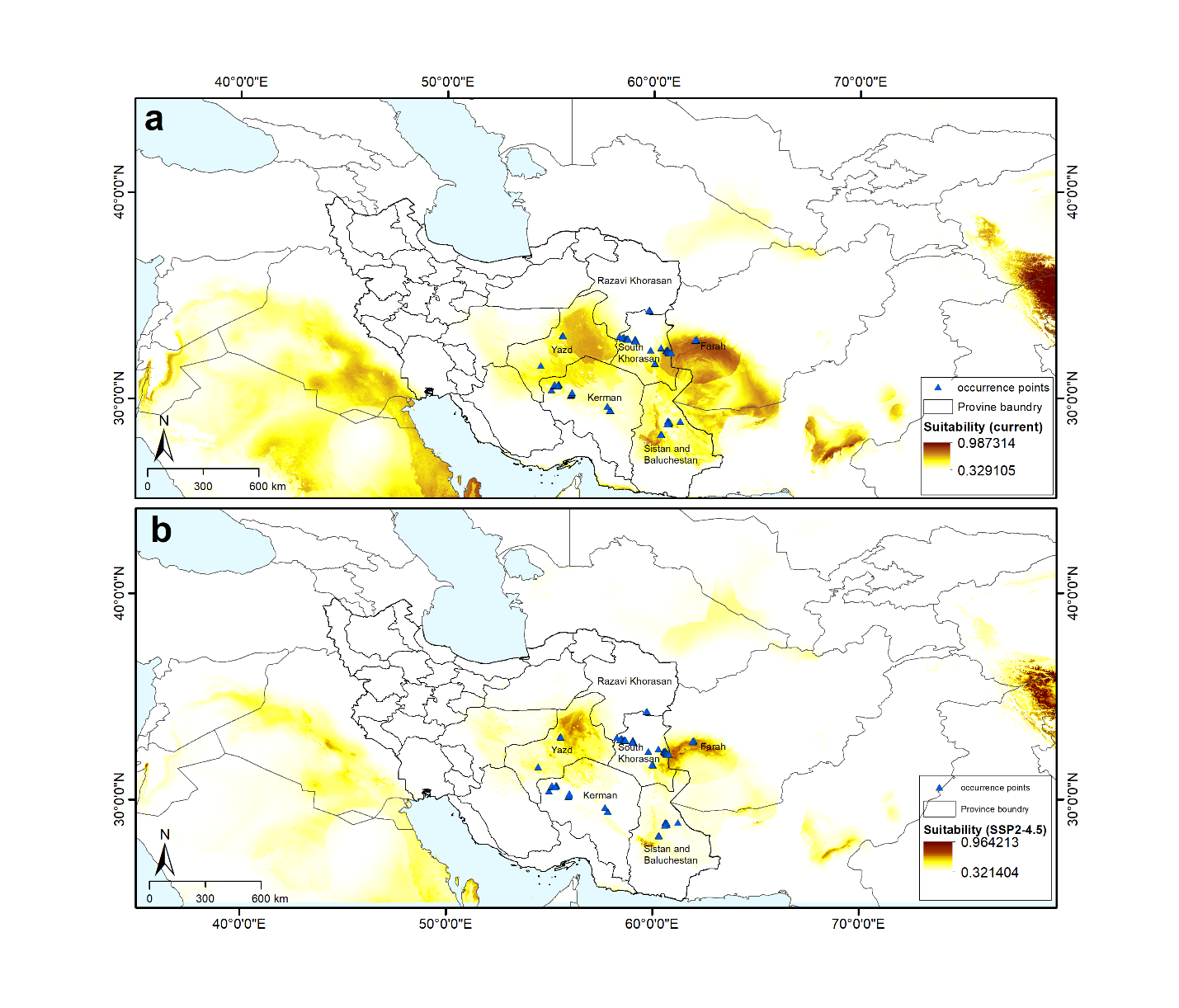


**Figure S2.** The major predictors of the suitability habitats of *Tamarix dubia*


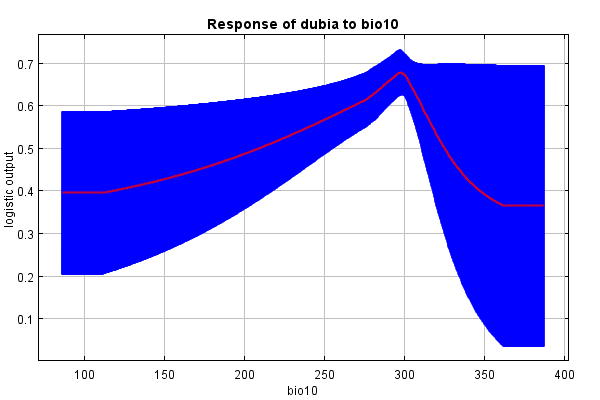

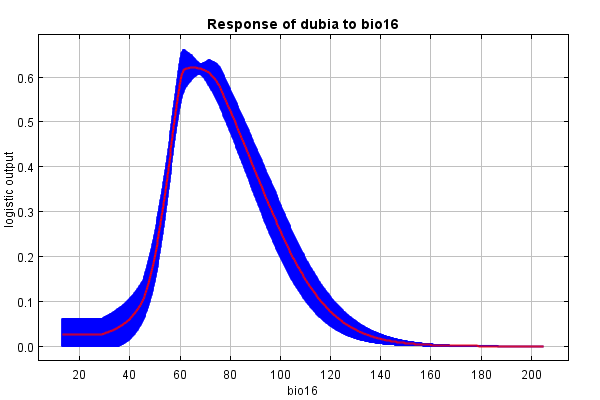


**Figure S3.** Gene flow and dispersal route among *Tamarix* *dubia* populations using RASP phytogeography tree based on binary MCMC model (Given Ijbari et al., 2024)


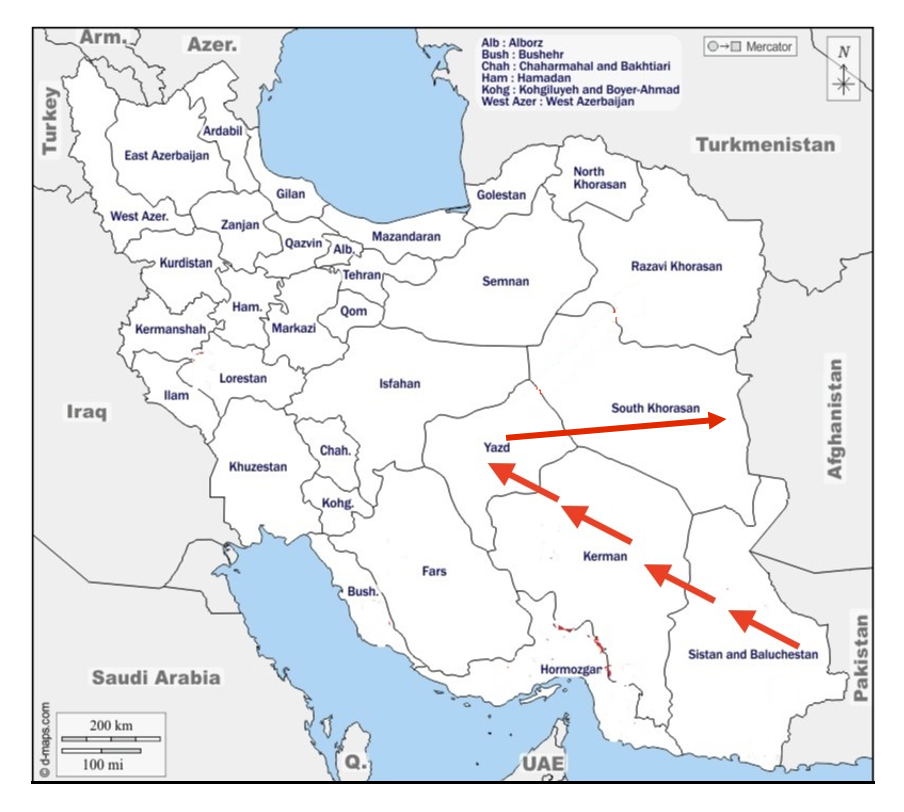

Supplement: Supplementary file 1 — Data S1. [file ECE3-15-e71877-s001.docx]
